# Nilutamide

**DOI:** 10.1107/S1600536812003728

**Published:** 2012-02-04

**Authors:** Niraj S. Trasi, Phillip E. Fanwick, Lynne S. Taylor

**Affiliations:** a Department of Industrial and Physical Pharmacy, Purdue University, West Lafayette, IN 47907, USA; b Department of Chemistry, Purdue University, West Lafayette, IN 47907, USA

## Abstract

The crystal structure of nilutamide [systematic name: 5,5-dimethyl-3-[4-nitro-3-(trifluoro­meth­yl)phen­yl]imidazolidine-2,4-dione], C_12_H_10_F_3_N_3_O_4_, was determined at 150 K. The dihedral angle between the mean planes through the imidazoline [maximum deviation = 0.0396 (14) Å] and benzene rings is 51.49 (5)°. The mol­ecule exhibits inter­molecular hydrogen bonding *via* N—H⋯O inter­actions, resulting in the formation of chains parallel to the *c* axis.

## Related literature

For the structure of a related compound, see: Cense *et al.* (1994 ▶).
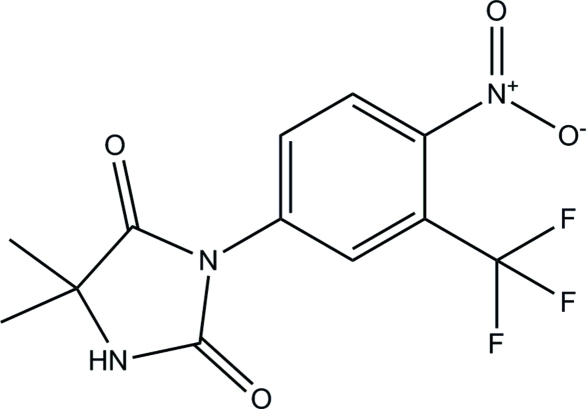


## Experimental

### 

#### Crystal data

C_12_H_10_F_3_N_3_O_4_
*M* *_r_* = 317.23
Monoclinic, 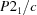

*a* = 12.3304 (9) Å
*b* = 9.8875 (2) Å
*c* = 12.2118 (3) Å
β = 117.322 (8)°
*V* = 1322.74 (14) Å^3^
*Z* = 4
Cu *K*α radiation
μ = 1.31 mm^−1^
*T* = 150 K
0.20 × 0.12 × 0.05 mm

#### Data collection

Rigaku Rapid II diffractometer
Absorption correction: multi-scan (*CrystalClear*; Rigaku, 2001 ▶) *T*_min_ = 0.733, *T*_max_ = 0.937
13145 measured reflections
2324 independent reflections
2019 reflections with *I* > 2σ(*I*)
*R*_int_ = 0.028

#### Refinement

*R*[*F* ^2^ > 2σ(*F* ^2^)] = 0.032
*wR*(*F* ^2^) = 0.085
*S* = 1.12
2324 reflections
206 parameters
H atoms treated by a mixture of independent and constrained refinement
Δρ_max_ = 0.31 e Å^−3^
Δρ_min_ = −0.22 e Å^−3^

### 

Data collection: *CrystalClear* (Rigaku, 2001 ▶); cell refinement: *CrystalClear*; data reduction: *CrystalClear*; program(s) used to solve structure: *SIR2004* (Burla *et al.*, 2005 ▶); program(s) used to refine structure: *SHELXL97* (Sheldrick, 2008 ▶) and a local program based on the method of Prince & Nicholson (1983 ▶); molecular graphics: *ORTEPII* (Johnson, 1976 ▶) and *PLATON* (Spek, 2009 ▶); software used to prepare material for publication: *SHELXL97* and local programs.

## Supplementary Material

Crystal structure: contains datablock(s) global, I. DOI: 10.1107/S1600536812003728/rz2702sup1.cif

Structure factors: contains datablock(s) I. DOI: 10.1107/S1600536812003728/rz2702Isup2.hkl

Supplementary material file. DOI: 10.1107/S1600536812003728/rz2702Isup3.mol

Supplementary material file. DOI: 10.1107/S1600536812003728/rz2702Isup4.cml

Additional supplementary materials:  crystallographic information; 3D view; checkCIF report

## Figures and Tables

**Table 1:** Hydrogen-bond geometry (Å, °)

*D*—H⋯*A*	*D*—H	H⋯*A*	*D*⋯*A*	*D*—H⋯*A*
N14—H14⋯O12^i^	0.84 (2)	2.06 (2)	2.894 (2)	172.3 (15)

Symmetry code: (i) 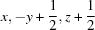.

**Fig. 1.**
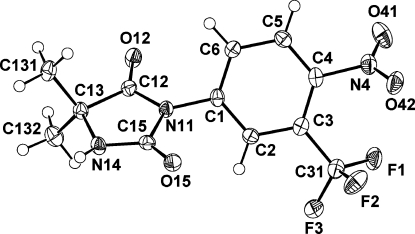
The molecular structure of the title compound indicating the 50% probability displacement ellipsoids and the atomic numbering for the non-H atoms.

## supplementary crystallographic information

### Comment

The title compound is a potent antiandrogen used primarily in the treatment of advanced stage prostrate cancer. While no single-crystal structure has been reported for this compound before, there have been reported structures for structurally related compounds like flutamide by Cense *et al.* (1994). However, the molecular arrangement of nilutamide is different from flutamide since flutamide does not exhibit any hydrogen bonding between the NH and the CO.

The imidazoline ring of the title compound (Fig. 1) is roughly planar [maximum deviation 0.0396 (14) Å for atom C15] and forms a dihedral angle of 51.49 (5)° with the benzene ring. The nitro group is tilted by 56.35 (7)° with respect to the benzene ring. In the crystal, molecules are linked by intermolecular N—H···O hydrogen interactions (Table 1) forming chains parallel to the *c* axis.

### Experimental

Nilutamide powder was obtained from Sigma-Aldrich Company and used without further purification.A solution of the compound (20 mg ml^-1^) was prepared in acetonitrile in a 3 ml glass vial. The solution was allowed to evaporate slowly by sealing the vial with parafilm and making a few small holes in the parafilm using a fine needle.

### Refinement

The imidazoline H atom was located in a difference Fourier map and refined freely [N–H = 0.841 (19) Å]. All other H atoms were positioned geometrically [C–H = 0.95–0.98 Å] and refined using a riding model, with *U*_iso_(H) = 1.2 *U*_eq_(C) or 1.5 *U*_eq_(C) for methyl H atoms. A rotating group model was applied to the methyl groups. Seven outliers (-2 0 2, -8 7 8, -8 2 13, -6 2 13, -9 1 14, -7 1 14, -6 1 14), were removed from the final refinement using a local program based on the method of Prince & Nicholson (1983).

### Crystal data

**Table:**

C_12_H_10_F_3_N_3_O_4_	*F*(000) = 648
*M**_r_* = 317.23	*D*_x_ = 1.593 Mg m^−^^3^
Monoclinic, *P*2_1_/*c*	Cu *K*α radiation, λ = 1.54184 Å
Hall symbol: -P 2ybc	Cell parameters from 13145 reflections
*a* = 12.3304 (9) Å	θ = 4–66°
*b* = 9.8875 (2) Å	µ = 1.31 mm^−^^1^
*c* = 12.2118 (3) Å	*T* = 150 K
β = 117.322 (8)°	Needle, colorless
*V* = 1322.74 (14) Å^3^	0.20 × 0.12 × 0.05 mm
*Z* = 4	

### Data collection

**Table:**

Rigaku Rapid II diffractometer	2019 reflections with *I* > 2σ(*I*)
Confocal optics monochromator	*R*_int_ = 0.028
ω scans	θ_max_ = 66.6°, θ_min_ = 4.0°
Absorption correction: multi-scan (*CrystalClear*; Rigaku, 2001)	*h* = −14→14
*T*_min_ = 0.733, *T*_max_ = 0.937	*k* = −11→11
13145 measured reflections	*l* = −13→14
2324 independent reflections	

### Refinement

**Table:**

Refinement on *F*^2^	H atoms treated by a mixture of independent and constrained refinement
Least-squares matrix: full	*w* = 1/[σ^2^(*F*_o_^2^) + (0.0389*P*)^2^ + 0.5381*P*] where *P* = (*F*_o_^2^ + 2*F*_c_^2^)/3
*R*[*F*^2^ > 2σ(*F*^2^)] = 0.032	(Δ/σ)_max_ < 0.001
*wR*(*F*^2^) = 0.085	Δρ_max_ = 0.31 e Å^−^^3^
*S* = 1.12	Δρ_min_ = −0.22 e Å^−^^3^
2324 reflections	Extinction correction: *SHELXL97* (Sheldrick 2008)
206 parameters	Extinction coefficient: 0.72E-02
0 restraints	

### Special details

**Table:**

Geometry. All e.s.d.'s (except the e.s.d. in the dihedral angle between two l.s. planes) are estimated using the full covariance matrix. The cell e.s.d.'s are taken into account individually in the estimation of e.s.d.'s in distances, angles and torsion angles; correlations between e.s.d.'s in cell parameters are only used when they are defined by crystal symmetry. An approximate (isotropic) treatment of cell e.s.d.'s is used for estimating e.s.d.'s involving l.s. planes.
Refinement. Outlier data were removed using a local program based on the method of Prince and Nicholson.Refinement on *F*^2^ for ALL reflections except for 0 with very negative *F*^2^ or flagged by the user for potential systematic errors. Weighted *R*-factors *wR* and all goodnesses of fit *S* are based on *F*^2^, conventional *R*-factors *R* are based on *F*, with *F* set to zero for negative *F*^2^. The observed criterion of *F*^2^ > σ(*F*^2^) is used only for calculating R_factor_obs *etc*. and is not relevant to the choice of reflections for refinement. *R*-factors based on *F*^2^ are statistically about twice as large as those based on *F*, and *R*- factors based on ALL data will be even larger.

### Fractional atomic coordinates and isotropic or equivalent isotropic displacement parameters (Å^2^)

**Table:**

	*x*	*y*	*z*	*U*_iso_*/*U*_eq_	
F1	0.23075 (8)	0.18106 (10)	0.90252 (9)	0.0335 (3)	
F2	0.15392 (8)	0.06052 (10)	0.99582 (10)	0.0362 (3)	
F3	0.26813 (8)	0.22939 (11)	1.08854 (9)	0.0414 (3)	
O12	−0.39601 (9)	0.28832 (12)	0.72787 (9)	0.0254 (3)	
O15	−0.12113 (9)	0.25489 (11)	1.13651 (9)	0.0236 (3)	
O41	0.14267 (12)	0.47236 (14)	0.74335 (12)	0.0419 (3)	
O42	0.26114 (10)	0.46290 (12)	0.94084 (12)	0.0350 (3)	
N4	0.16293 (12)	0.44744 (13)	0.84896 (13)	0.0264 (3)	
N11	−0.23339 (10)	0.27413 (13)	0.92377 (11)	0.0189 (3)	
N14	−0.32753 (11)	0.20473 (13)	1.03076 (12)	0.0212 (3)	
C1	−0.13395 (12)	0.31698 (15)	0.90267 (13)	0.0189 (3)	
C2	−0.03031 (13)	0.23688 (15)	0.94671 (13)	0.0196 (3)	
C3	0.06962 (13)	0.27696 (15)	0.93109 (13)	0.0199 (3)	
C4	0.06014 (13)	0.39789 (15)	0.86842 (13)	0.0208 (3)	
C5	−0.04267 (13)	0.47766 (16)	0.82365 (13)	0.0214 (3)	
C6	−0.14103 (13)	0.43759 (15)	0.84249 (13)	0.0198 (3)	
C12	−0.35389 (13)	0.26571 (15)	0.83743 (13)	0.0187 (3)	
C13	−0.42547 (13)	0.22238 (15)	0.90554 (13)	0.0197 (3)	
C15	−0.21768 (13)	0.24400 (14)	1.04444 (13)	0.0187 (3)	
C31	0.18088 (13)	0.18783 (16)	0.98009 (15)	0.0261 (4)	
C131	−0.51338 (13)	0.33459 (17)	0.89947 (15)	0.0259 (4)	
C132	−0.49281 (15)	0.08983 (17)	0.85113 (15)	0.0289 (4)	
H2	−0.0276	0.1543	0.9877	0.023*	
H5	−0.0465	0.5589	0.7805	0.026*	
H6	−0.2121	0.4924	0.8144	0.024*	
H14	−0.3407 (15)	0.2040 (18)	1.0925 (17)	0.025 (5)*	
H13A	−0.5518	0.3103	0.9514	0.039*	
H13B	−0.5765	0.3459	0.8141	0.039*	
H13C	−0.4682	0.4195	0.9290	0.039*	
H13D	−0.4334	0.0188	0.8615	0.043*	
H13E	−0.5479	0.1023	0.7632	0.043*	
H13F	−0.5402	0.0635	0.8938	0.043*	

### Atomic displacement parameters (Å^2^)

**Table:**

	*U*^11^	*U*^22^	*U*^33^	*U*^12^	*U*^13^	*U*^23^
F1	0.0288 (5)	0.0330 (6)	0.0494 (6)	0.0026 (4)	0.0270 (5)	−0.0030 (4)
F2	0.0316 (5)	0.0277 (5)	0.0541 (7)	0.0076 (4)	0.0239 (5)	0.0092 (5)
F3	0.0242 (5)	0.0495 (7)	0.0348 (6)	0.0072 (4)	0.0000 (4)	−0.0041 (5)
O12	0.0208 (5)	0.0387 (7)	0.0175 (6)	0.0017 (5)	0.0096 (5)	0.0017 (5)
O15	0.0213 (5)	0.0300 (6)	0.0169 (6)	0.0000 (4)	0.0065 (5)	0.0026 (4)
O41	0.0498 (8)	0.0485 (8)	0.0436 (8)	−0.0091 (6)	0.0354 (7)	−0.0007 (6)
O42	0.0190 (6)	0.0307 (7)	0.0522 (8)	−0.0037 (5)	0.0138 (6)	−0.0056 (6)
N4	0.0255 (7)	0.0240 (7)	0.0356 (8)	−0.0028 (5)	0.0190 (6)	−0.0046 (6)
N11	0.0165 (6)	0.0242 (7)	0.0173 (6)	0.0004 (5)	0.0088 (5)	0.0011 (5)
N14	0.0209 (6)	0.0295 (7)	0.0163 (6)	−0.0012 (5)	0.0111 (5)	0.0011 (5)
C1	0.0176 (7)	0.0244 (8)	0.0165 (7)	−0.0024 (6)	0.0093 (6)	−0.0028 (6)
C2	0.0214 (7)	0.0198 (8)	0.0180 (7)	−0.0003 (6)	0.0095 (6)	0.0001 (6)
C3	0.0177 (7)	0.0238 (8)	0.0172 (7)	0.0000 (6)	0.0072 (6)	−0.0041 (6)
C4	0.0184 (7)	0.0261 (8)	0.0198 (7)	−0.0050 (6)	0.0104 (6)	−0.0053 (6)
C5	0.0240 (7)	0.0217 (8)	0.0186 (7)	−0.0026 (6)	0.0098 (6)	−0.0003 (6)
C6	0.0186 (7)	0.0230 (8)	0.0177 (7)	0.0014 (6)	0.0081 (6)	−0.0009 (6)
C12	0.0197 (7)	0.0196 (8)	0.0182 (7)	0.0016 (6)	0.0099 (6)	−0.0006 (6)
C13	0.0187 (7)	0.0254 (8)	0.0166 (7)	−0.0011 (6)	0.0093 (6)	−0.0002 (6)
C15	0.0225 (8)	0.0169 (7)	0.0187 (7)	0.0021 (6)	0.0112 (6)	0.0013 (6)
C31	0.0211 (8)	0.0273 (9)	0.0298 (9)	−0.0004 (6)	0.0118 (7)	−0.0022 (7)
C131	0.0213 (8)	0.0336 (9)	0.0264 (8)	0.0016 (7)	0.0142 (7)	−0.0022 (7)
C132	0.0291 (8)	0.0289 (9)	0.0289 (9)	−0.0069 (7)	0.0135 (7)	−0.0027 (7)

### Geometric parameters (Å, º)

**Table:**

F1—C31	1.3470 (18)	C2—H2	0.9500
F2—C31	1.3381 (19)	C3—C4	1.395 (2)
F3—C31	1.3313 (19)	C3—C31	1.504 (2)
O12—C12	1.2133 (18)	C4—C5	1.375 (2)
O15—C15	1.2101 (18)	C5—C6	1.392 (2)
O41—N4	1.2210 (18)	C5—H5	0.9500
O42—N4	1.2254 (18)	C6—H6	0.9500
N4—C4	1.4762 (18)	C12—C13	1.5258 (19)
N11—C12	1.3740 (18)	C13—C131	1.529 (2)
N11—C15	1.4268 (18)	C13—C132	1.530 (2)
N11—C1	1.4281 (17)	C131—H13A	0.9800
N14—C15	1.3437 (18)	C131—H13B	0.9800
N14—C13	1.4602 (19)	C131—H13C	0.9800
N14—H14	0.841 (19)	C132—H13D	0.9800
C1—C6	1.382 (2)	C132—H13E	0.9800
C1—C2	1.385 (2)	C132—H13F	0.9800
C2—C3	1.388 (2)		
			
O41—N4—O42	125.39 (13)	N11—C12—C13	106.92 (12)
O41—N4—C4	117.55 (13)	N14—C13—C12	101.37 (11)
O42—N4—C4	117.04 (13)	N14—C13—C131	111.39 (12)
C12—N11—C15	111.48 (11)	C12—C13—C131	110.15 (12)
C12—N11—C1	126.52 (12)	N14—C13—C132	112.05 (13)
C15—N11—C1	121.83 (12)	C12—C13—C132	109.76 (12)
C15—N14—C13	113.43 (12)	C131—C13—C132	111.66 (12)
C15—N14—H14	119.3 (12)	O15—C15—N14	130.31 (14)
C13—N14—H14	122.2 (12)	O15—C15—N11	123.39 (13)
C6—C1—C2	121.44 (13)	N14—C15—N11	106.30 (12)
C6—C1—N11	120.12 (13)	F3—C31—F2	106.75 (13)
C2—C1—N11	118.41 (13)	F3—C31—F1	107.08 (12)
C1—C2—C3	120.15 (14)	F2—C31—F1	106.13 (12)
C1—C2—H2	119.90	F3—C31—C3	112.82 (13)
C3—C2—H2	119.90	F2—C31—C3	111.52 (12)
C2—C3—C4	117.63 (13)	F1—C31—C3	112.13 (13)
C2—C3—C31	118.88 (14)	C13—C131—H13A	109.50
C4—C3—C31	123.48 (13)	C13—C131—H13B	109.50
C5—C4—C3	122.64 (13)	H13A—C131—H13B	109.50
C5—C4—N4	116.64 (13)	C13—C131—H13C	109.50
C3—C4—N4	120.71 (13)	H13A—C131—H13C	109.50
C4—C5—C6	119.02 (14)	H13B—C131—H13C	109.50
C4—C5—H5	120.50	C13—C132—H13D	109.50
C6—C5—H5	120.50	C13—C132—H13E	109.50
C1—C6—C5	119.09 (13)	H13D—C132—H13E	109.50
C1—C6—H6	120.50	C13—C132—H13F	109.50
C5—C6—H6	120.50	H13D—C132—H13F	109.50
O12—C12—N11	126.85 (13)	H13E—C132—H13F	109.50
O12—C12—C13	126.22 (13)		
			
C12—N11—C1—C6	51.1 (2)	C15—N11—C12—C13	−1.60 (16)
C15—N11—C1—C6	−123.91 (15)	C1—N11—C12—C13	−177.03 (13)
C12—N11—C1—C2	−130.60 (15)	C15—N14—C13—C12	6.36 (16)
C15—N11—C1—C2	54.39 (19)	C15—N14—C13—C131	−110.78 (14)
C6—C1—C2—C3	0.1 (2)	C15—N14—C13—C132	123.33 (14)
N11—C1—C2—C3	−178.20 (13)	O12—C12—C13—N14	177.45 (14)
C1—C2—C3—C4	−1.1 (2)	N11—C12—C13—N14	−2.59 (15)
C1—C2—C3—C31	179.93 (13)	O12—C12—C13—C131	−64.52 (19)
C2—C3—C4—C5	0.7 (2)	N11—C12—C13—C131	115.44 (13)
C31—C3—C4—C5	179.61 (14)	O12—C12—C13—C132	58.8 (2)
C2—C3—C4—N4	179.62 (13)	N11—C12—C13—C132	−121.22 (13)
C31—C3—C4—N4	−1.4 (2)	C13—N14—C15—O15	172.23 (15)
O41—N4—C4—C5	−55.63 (19)	C13—N14—C15—N11	−7.49 (16)
O42—N4—C4—C5	122.84 (15)	C12—N11—C15—O15	−174.20 (14)
O41—N4—C4—C3	125.35 (16)	C1—N11—C15—O15	1.5 (2)
O42—N4—C4—C3	−56.18 (19)	C12—N11—C15—N14	5.55 (16)
C3—C4—C5—C6	0.8 (2)	C1—N11—C15—N14	−178.77 (13)
N4—C4—C5—C6	−178.24 (13)	C2—C3—C31—F3	−99.05 (16)
C2—C1—C6—C5	1.4 (2)	C4—C3—C31—F3	82.01 (18)
N11—C1—C6—C5	179.61 (13)	C2—C3—C31—F2	21.1 (2)
C4—C5—C6—C1	−1.8 (2)	C4—C3—C31—F2	−157.86 (14)
C15—N11—C12—O12	178.37 (15)	C2—C3—C31—F1	139.95 (14)
C1—N11—C12—O12	2.9 (2)	C4—C3—C31—F1	−39.0 (2)

### Hydrogen-bond geometry (Å, º)

**Table:**

*D*—H···*A*	*D*—H	H···*A*	*D*···*A*	*D*—H···*A*
N14—H14···O12^i^	0.84 (2)	2.06 (2)	2.894 (2)	172.3 (15)

Symmetry code: (i) *x*, −*y*+1/2, *z*+1/2.
